# Consumers’ Preferences for Chicken Fed on Different Processed Animal Proteins: A Best–Worst Analysis in Italy

**DOI:** 10.3390/nu15071800

**Published:** 2023-04-06

**Authors:** Mario Amato, Eugenio Demartini, Anna Gaviglio, Maria Elena Marescotti, Fabio Verneau

**Affiliations:** 1 Department of Political Sciences, University of Napoli Federico II, Via Rodinò 22, 80138 Napoli, Italy; 2 Department of Veterinary Medicine and Animal Sciences (DIVAS), University of Milan, Via dell’Università, 6, 26900 Lodi, Italy

**Keywords:** consumer, preferences, acceptance, best–worst analysis, chicken, protein, feed, feed meal, insect, pork, poultry, regulations, processed animal proteins

## Abstract

The increase in meat consumption expected in the next decade will require more and more proteins for animal feeding. The recent amendments to the European “BSE Regulation” allow the use of insects and porcine-based meals in poultry farming, providing novel, sustainable substitutes for vegetable fodder. While the technological and nutritional properties of novel feeds containing processed animal proteins are widely recognized, far less is known about consumers’ acceptance of meat produced by animals fed on animal-based meals. In the present research, a best–worst survey was applied to estimate consumers’ preferences for chicken fed on plants, insects, or porcine-based meals using a sample of 205 Italian consumers. Furthermore, product price, type of farming, and “Free-from” labeling were considered in the analysis to evaluate the relative importance of feed ingredients compared to other important attributes of meats. The results show that the most relevant attributes are type of farming and “Free-from” claims, while type of feed represents the third attribute in order of importance. Notably, both insect and porcine flour are considered as negative characteristics of the product, suggesting that mandatory labeling signaling the use of these feeds would negatively impact on the value of chicken meat.

## 1. Introduction

Despite relevant differences between most affluent and developing countries [1,2], meat consumption will increase in the next decade due to world population growth [3]. This trend is causing public concern from several points of view. Among the most relevant issues, some authors emphasize that to increase the number of farmed animals, more and more high-quality proteins will be needed soon for animal feeding [4,5]. Plant-based meal represents the most important source of protein for livestock production, being the only type of protein allowed for ruminants’ nutrition [6]. The transformation of natural areas into arable land to respond to the new market request [7,8] and the increase in competition for the use of land for feed or food [9] are not viable options to meet sustainable development goals; thus, alternative proteins need to be introduced in animal diets [10], unless disruptive technologies will be found to significantly increase crop yields.

Some substitutes of plant-based meals have been proposed in the last two decades, such as microalgae [11] and processed municipal waste [12]; however, the most efficient approach is the use of processed animal proteins (PAP) derived from farmed animal by-products [13] and insects [4,14]. Unfortunately, food safety regulations have hampered the use of PAP for many years [5,15]. In the European Union, the Regulation (EC) No 999/2001, also known as the ‘BSE Regulation’, prohibits the use of animal-based meals for animal rearing in response to the Bovine Spongiform Encephalopathy epidemic. In recent years, two amendments to the BSE Regulation have been approved that permit the use of insects as feed in aquaculture since 2017 [16] and in poultry and pigs since 2021 [17]. Furthermore, the latter amendment allows the use of ‘PAP derived from porcine animals’ in poultry and ‘PAP derived from poultry’ in porcine farming, respectively. Hence, starting from 2021, European poultry and pig farmers can benefit from the reintroduction of PAP in animal diets to replace part of plant-based proteins.

While many studies focused on the economic and technical implications of the use of PAP as feedstuffs, far less attention has been devoted to consumers’ acceptance of this innovation to evaluate the market potential of meat produced from animals fed on this type of proteins [18]. The reminiscence of the BSE scandal and animal welfare concern might induce negative attitudes toward the use of porcine and poultry by-products as feedstuffs, and the sense of disgust toward insects might represent an obstacle to the introduction of this feed in the livestock sector [18,19]. In this regard, as noted by Altmann et al. [10], the introduction of insects and animal by-products products might raise the attention of different stakeholders and stimulate the request of mandatory labeling on type of feedstuffs used in fish, poultry, and pig farming, as recently occurred with vegan and meat-sounding labeling [20].

### 1.1. European Consumers’ Attitudes toward the Use of Processed Animal Protein Feeds

The literature on European consumers’ attitudes toward insect-based fed shows some clear evidence. One of the first survey on this topic was conducted in Belgium [21] and found that both farmers and consumers possess positive beliefs and attitudes toward the use of insects as feedstuffs, and that they would eat meat produced by animals fed on insects. However, this study reported that Belgian consumers would not eat foods containing insects. Similar results are reported in studies conducted in other European countries, such as Italy [22], Poland [23], the Netherlands [24,25], Germany [25], Greece [26], and Portugal [27], suggesting that while consumers refuse entomophagy [28,29], indirect entomophagy, i.e., eating something that ate insects, does not represent a real barrier for the introduction of insects as feed in the livestock sector. With regard to the type of food considered in research, some authors specifically focused on one type of meat from animals fed on insect meals, such as poultry [10,18,19,24,30] or farmed fish [26,31,32,33], while others generically explored the acceptance of “meats from animals fed on insects” [25,34]. Finally, some studies considered different species, offering comparisons between poultry, pork, and/or beef [21,23,27] and showed that the acceptance of insects as feedstuff does not depend on the considered species. Furthermore, since insects as feed represent an innovation in the food supply chain, some authors hypothesized that consumers might not be aware of the benefits of feeding animal on PAP based on insects and tested the role of information on product acceptance. In this case, the literature does not offer clear results. For instance, information on environmental and nutritional benefits of insect-based meal was able to increase consumers’ willingness to pay for duck (note that these results are reported in the published papers and information derives from direct contact with the authors) and chicken meat in Sogari et al. [19] and Altmann et al. [10], respectively; however, consumers’ opinions were not influenced by information for most of the products evaluated in Laureati et al. [22], Menozzi et al. [18], and Naranjo-Guevara et al. [25]. Unfortunately, while the literature on consumers’ acceptance of meat from animals fed on insects has reached a good amount of contribution in recent years, we are not aware of any research focusing on consumers’ opinions for meat from animals fed on poultry or porcine by-product meals. Nonetheless, as European consumers might be reluctant regarding the use of farmed animal by-products as feed, there is an urgent need to explore their preferences for poultry/pork from animals fed on porcine- or poultry-based proteins, respectively.

### 1.2. Aims of the Research

Building on previous studies, the present research aims to contribute to the literature on consumers’ acceptance of PAP as feedstuffs in two ways. Firstly, we measured preferences for the use of both insects and porcine-based feedstuffs in chicken farming. This will provide a first estimate of the effects of a mandatory labeling scheme on feed provision at the farm level, including all PAP allowed by European regulations. Secondly, we measured the relevance attached by consumers to feeding characteristics as opposed to other important extrinsic factors, namely rearing conditions, price and the presence of clean labels to communicate specific “Free-from” claims. This will provide relevant information on consumers’ demand for improved characteristics of chicken meat, thereby suggesting useful elements to policymakers interested in food labeling schemes, addressing communication about PAP in poultry feeding, and discussing the challenges and risks that this innovation might pose in terms of marketing and competition strategies.

To meet the research objectives, an online questionnaire was distributed in Italy. A best–worst analysis was used to estimate consumers’ preferences for a 400 g package of chicken breast characterized by different types of feed provision (vegetable fodder, pork flour, and insect flour), farming system (intensive, extensive indoor, and extensive outdoor), price (EUR 3.10, EUR 4.50, and EUR 5.90), and “Free-from” labeling (antibiotics, genetically modified organism—GMO, and cruelty free).

## 2. Materials and Methods

### 2.1. Data Collection

To answer the research questions, an online survey was implemented via the Qualtrics software (www.qualtrics.com) and conducted during February 2022. The information presented here represents part of a wider data collection process aimed at exploring Italian consumers’ perception, attitudes, and knowledge of sustainable poultry farming. Participants were invited and recruited through snowball sampling, a non-probabilistic sampling technique, via online advertisements on the authors’ social media platforms. The selection criteria for participants included being at least 18 years old and being a meat eater; vegetarians or vegans were excluded from the study as the questionnaire surveyed participants regarding their most and least preferred attributes when buying chicken meat. After filling out an informed consent form, a concise instruction note explained to the participants that they were to look at nine variations of the same product and choose which attribute was their most favorite and which was their least favorite attribute for each product variation. The survey also included items aimed at evaluating consumers’ perception, attitudes, and preferences toward insect- and pork-based flour used as feed for poultry, and sociodemographic characteristics, with the former being part of a wider study and not disclosed in this paper. The average time for completing the questionnaire was 15 min; therefore, after carefully checking and excluding “speeders” (participants who completed the questionnaire in less than 5 min), the final sample consisted of 205 correctly compiled responses; the sample is composed of 58.54% of females, with a mean age of 40.09 years (SD = 15.84) and mostly residents in the North-Western part of Italy (43.02%) with at least upper secondary education (49.75%). Table 1 summarizes the main sociodemographic characteristics of the collected sample.

### 2.2. Best–Worst Scaling for Consumers’ Preference Estimation

To estimate consumers’ preferences for different attributes of chicken meat, the best–worst scaling (BWS) approach was applied. The BWS approach is a method of measuring the relative importance of or preference for different attributes of a product or service. This survey technique involves presenting participants with a set of profiles of a targeted product, with each of this set being composed of different levels of a set of attributes established by the researchers, and respondents are required to state their most and least preferred levels of attributes among the presented profile [35,36]. The BWS method assumes that the most and least preferred options are more indicative of the underlying preferences than the intermediate options; by comparing the most and least preferred options, the BWS method aims to extract the maximum amount of information about consumers’ underlying preferences. This provides more accurate and reliable results compared to other scaling methods with some limitations, such as attribute impact measurements that are confounded with level scale values in discrete choice experimentation and subjective scale measurements resulting from rating-based methods [37,38]. In fact, the BWS method has been found to be useful in various research fields, such as marketing [39], environmental economics [40], transportation [41], and health economics [41].

Furthermore, the BWS method allows the measurement of the maximum difference (maxdiff) between attribute levels on a common utility scale and can be developed in three different ways: object case, profile case, and multi-profile case. The present work focused on the profile case, also known as BWS Case 2 [42], in which a product is described by a common set of attributes and levels, called profiles, which are presented one at a time. These profiles are created using experimental designs, where the attributes are fixed but the combination of attribute levels in each profile varies. Participants are presented with one profile at a time and are asked to select the best and worst attribute-levels among the options presented to them, which is different from traditional conjoint analysis (CA) and discrete choice experiments (DCE). This process is repeated until all the profiles have been evaluated.

### 2.3. Product and Attribute Selection

The product used in the BWS questions was a 400 g package of chicken breast. To determine the attributes corresponding to consumers’ interest given the product used in the BWS questions, a discussion about the topic was organized within the research team, followed by consultations with experts on the field (poultry breeders, retail managers, and researchers on livestock feeding). Several extrinsic attributes that could be communicated via labeling emerged during the discussion and consultations. As part of price, the most cited attributes were (a) type of rearing; (b) animal welfare; (c) feed characteristics, (d) presence/absence of antibiotics, and (e) organic certification. The attributes and levels identified as most important were then classified to construct the best–worst profiles. The attributes and corresponding levels finally chosen for the survey are presented in Table 2.

**Figure 1:**
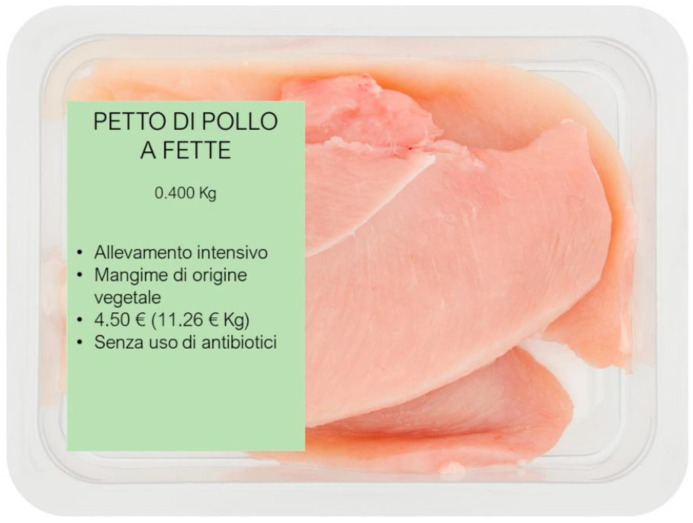
One of the product’s profiles presented to participants.

The attribute “*Type of feeding*” is crucial to the aim of the present research because it explicitly refers to the use of PAP in poultry farming as allowed by the new amendments of the BSE Regulation [17]. Accordingly, along with the level concerning the exclusive use of plant-based meals, two further levels were considered, namely feeds supplemented with protein extracted from insects or protein produced with porcine by-products. The second attribute considered was the “*Type of rearing*”, which has been previously found as a driver of consumers’ food preferences [43,44]. In this case, the three levels qualifying the attribute were intensive rearing system, extensive indoor rearing system (semi-intensive), and extensive outdoor rearing system (free-range). The attribute “*Price*” was also considered in the analysis. The three price levels were identified by considering the sales prices for the 400 g package of chicken breast at different distribution outlets in the two cities of Naples and Milan (Italy). The average price (EUR 4.50; 11.26 EUR/kg), calculated considering all the references identified, represents the middle level. The other two levels (low price: EUR 3.10 at 7.74 EUR/kg, and high price: EUR 5.90 at 14.76 EUR/kg) were calculated by subtracting and adding a standard deviation to the average price, respectively. The last attribute was the “*Free-from*” labeling, which was represented by antibiotics, GMO, and cruelty-free levels. The importance of clean labels and the preference accorded by consumers for products with “Free-from” claims have also emerged clearly from the analysis of the specific literature [45,46]. Considering that each profile had 4 attributes (K) and each attribute had 3 levels (L), a 3^4^ (L^k^) orthogonal main-effect design was used to construct the profiles, which resulted in 9 different profiles. A profile example is provided in Figure 1.

### 2.4. Model Specification

Consumers’ preferences can be estimated from responses obtained from the BWS using different approaches, primarily using either a counting or a modeling approach. The first method involves determining scores that stand for respondents’ preferences by calculating the frequency of attribute-level *I* selected as the best (*B_in_*) and worst (*W_in_*) among all the questions for respondent *n*, as follows:

$${BW}_{in}=B_{in}-W_{in}$$

The second approach uses marginal or paired methods, which can also be analyzed at either the sample or respondent level [47,48,49]. Marginal models aggregate best–worst pairs to determine choice frequencies [49] but might lead to larger standard errors [47]; on the contrary, the paired estimation approach treat each best–worst pair as a choice outcome. Considering the approximation given by marginal models, in the present work, we opted for a paired estimation approach using a conditional logit analysis estimated by using the R software [50], following the guidelines of Aizaki and Fogarty [51]. A paired model postulates that each respondent chooses an attribute level as the best and another attribute level as the worst because this specific difference in utility represents the greatest utility difference among the other options. As each profile contains 4 items (K), we would have K(K−1) = 12 possible best–worst alternatives that each person could choose. Therefore, the probability of choosing an attribute level *b* as the best and an attribute level *w* (with *b* different from *w*) as the worst in a profile C of attribute levels *kl* can be conveyed via a conditional logit model with the systematic component of utility *u*, as follows:

$$\mathrm{Pr}\left(best=b,worst=w\right)=\frac{exp(u_{w}-u_{b})}{\Sigma_{k,l\subset C,k\neq l}exp(u_{l}-u_{k})}$$

In model estimation, an arbitrary attribute variable is omitted [51], and the coefficient of the omitted attribute variable is normalized to 0. In our case, the attribute “type of rearing” is the base attribute and is thus omitted from the model. Level variables are, instead, effect-coded variables, taking the value of −1 to indicate the base category. In our model, “intensive” rearing, “vegetable” feeding, the average price (“4.50”), and the claim of free from “antibiotics” are the base categories for each attribute. Considering the above, the systematic component of the utility of selecting alternative *b* $\left(u_{b}\right)$ is composed by both attribute and level variables and can be written as follows:

$$u_{b}=\beta_{1}Feeding+\beta_{2}Price+\beta_{3}Freefrom+\beta_{4}ExtIndoor+\beta_{5}ExtOutdoor{+\beta }_{6}Pork+\beta_{7}Insects\;+\beta_{8}Lowprice+\beta_{9}Highprice+\beta_{10}GMO+\beta_{11}Cruelty$$

where “*Feeding*”, “*Price*”, and “*Freefrom*” are attribute variables related to the three categories of attributes chosen; “*ExtIndoor*” and “*ExtOutdoor*” are level variables related to the “type of rearing” attribute; “*Pork*” and “*Insects*” are level variables related to the “type of feeding” attribute; “*Lowprice*” and “*Highprice*” are level variables related to the “*price*” attribute; and, lastly, “*GMO*” and “*Cruelty*” are level variables related to “*Freefrom*” attribute.

## 3. Results

### 3.1. Best–Worst Scoring

**Figure 2:**
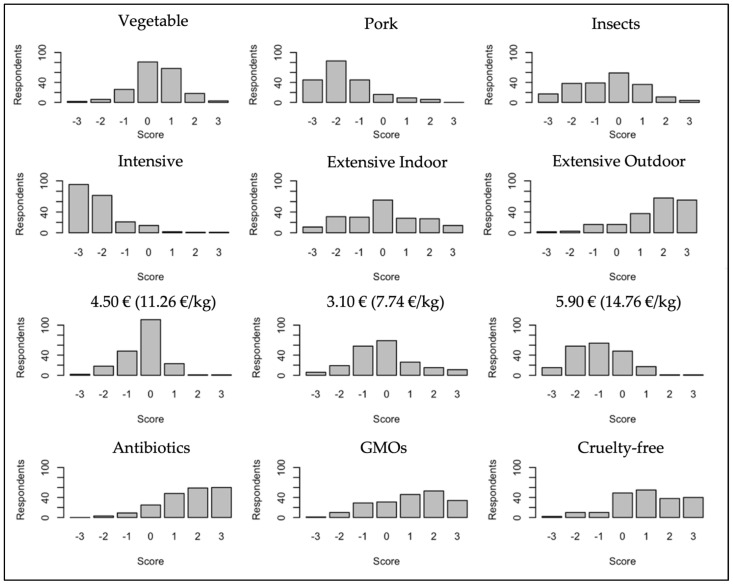
Distributions of best-minus-worst (BW) scores for the 12 levels.

The results of the BW scoring and the distribution of BWS scores are presented in Table 3 and Figure 2, respectively. The most preferred attribute level is extensive outdoor rearing, followed by the three levels related to “Free-from” labeling, namely the absence of antibiotics, the cruelty-free claim, and the absence of GMO (in descending order). On the contrary, the least preferred attribute level is intensive rearing (−437), followed by using pork flour (−325) and the highest price of EUR 5.90 for the 400 g chicken breast package (−203).

Among the remaining levels representing extensive rearing systems, extensive outdoor rearing is preferred (332) over extensive indoor rearing (−1). No clear difference can be seen among the three levels of the “Free-from” attribute, which are all positively evaluated by the respondents (331 for antibiotics, 202 for OGM, and 215 for cruelty free). Considering the attribute related to the type of feeding, using pork flour is the least preferred (−325), followed by insect meals (−96), while conventional vegetable feeding obtains a slightly positive valuation (69). In a similar fashion, the lower price of 3.10 EUR is also slightly preferred (−25) over the average price of EUR 4.50 (−62), while the highest price of EUR 5.90 gets the worst BW scoring (−203).

### 3.2. Conditional Logit Estimation

In the conditional logit estimation, the dependent variable is the utility that each respondent derives from choosing the best alternative in a profile of alternatives. The results shown in Table 3 suggest that several independent variables have a significant effect on consumers’ preferences toward the 400 g package of chicken meat. The estimated coefficients for the attribute variables shown in Table 4 represent the average impact of each considered attribute, i.e., the mean utility across all levels of an attribute [47].

In our case, the type of rearing is considered a base attribute; therefore, the attribute “Free-from” provides the highest attribute impact. On the other hand, the feeding type and price are considered less important by the respondents. In regard to the different types of feed considered at the level scale, it is worth pointing out that the presence of the level “pork flour” leads to a decrease of 64% (0.365 odds ratio) in the utility of choosing the product. On the other hand, the level “insect flour” has a non-significant, although slightly positive, effect on the utility of choosing the best alternative.

**Figure 3:**
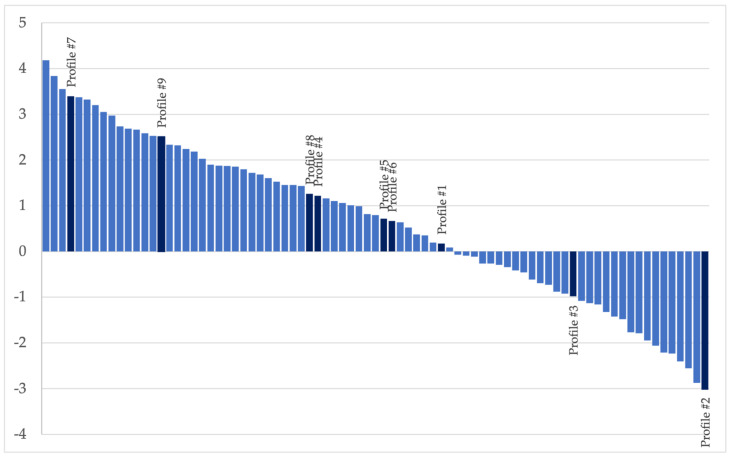
Distribution of the utility of each product profile included (dark blue) vs. not included in the survey (light blue).

Furthermore, Figure 3 represents a scenario analysis based on the estimates obtained in the present survey.

Specifically, the responses collected on the product profiles (Profile #1 to #9 in Figure 3) were firstly used to estimate the coefficients of consumers’ utility and then employed to predict consumers’ preferences on the profiles that were not included in the questionnaire. The nine profiles shown to the participants are highlighted and ranked in Table 5. The highest ranked profile among the profiles shown to the participants is a combination of extensive outdoor rearing, poultry fed on vegetable fodder, free from GMO, and carrying a price of 3.10 euros. On the other hand, the last ranked profile is chicken breast from animals reared intensively and with pork flour as fodder, free from GMO, and with a cost of 5.90 euros.

## 4. Discussion

The results of the present study contribute in several ways to the scientific knowledge about consumers’ preferences toward extrinsic attributes and food labels in the case of chicken breast from animals reared with alternative feeds. The main results highlight that the most relevant attributes are the type of farming and the “Free-from” claim on the label. In particular, the type of farming was selected by the respondents in more than 31% of the cases, and the “Free-from” attribute in almost 29% of the cases. This means that the level of salience is higher than in the cases of the other two attributes, feed (22.5%) and price (18%). The importance attached to the first two attributes is in line with previous findings. Using a conjoint approach, Martinez and colleagues [52] showed that type of farming is the most important attribute influencing consumer choice in the case of chicken. In the same fashion, a free-range claim is the most appealing in a choice experiment conducted on chicken breasts [53]. Finally, the relevance of the animal husbandry system also clearly emerges in a meta-analysis on consumer studies, with consumers perceiving the aspects of outdoor access, stocking density, and floor type among the most relevant selection factors [54].

The second attribute selected by the respondents is the presence of a “Free-from” claim on the label. These products should appeal to consumers for whom an ingredient is perceived to be detrimental. The use of “Free-from” claims can be interpreted as an example of clean labeling, which is one of the most interesting trends in firms’ communication strategies toward consumers [45,46].

The third attribute in order of importance is the feed used. In this case, the three investigated elements (vegetable fodder, pork flour, and insect flour) were selected as the best or worst option in only 22.5% of the cases, showing a lower salience of this attribute.

Finally, price was selected as the best or worst attribute in only 17% of the cases. Although price in many studies stands out as a very important element of choice, as Lusk [55] argued, there is a non-trivial minority of consumers who are relatively unconcerned about chicken prices, and these consumers are the target market for producers making strategic use of clean labels and other FOP (front of package) information. Therefore, when such attributes are present, especially in our case, low prices may attract less consumer attention.

Further and more insightful considerations can be made considering the conditional logit model. The results are substantially in line with those already discussed in the case of best–worst scoring. The attributes with the smallest impact on average utility are those of price and type of feed, while those with the greatest impact are the type of rearing and, above all, the presence of a “Free-from” claim. In other words, the attribute with the greatest salience is the type of farming because its elements are the ones most chosen as the best or worst and, therefore, most likely to have caught the attention of the respondents. Nevertheless, the attribute generating the greatest contribution in terms of average utility is the presence of a “Free-from” claim. 

However, when considering the results of the specific levels related to the type of feeding, two further interesting results emerge. First, both insect- and pork-based flours obtain a negative best–worst scoring. Yet, protein flours containing pork are rated much more negatively, as also evidenced in Table 2 and Figure 2 showing the distributions of the BWS scores. Second, the conditional logit estimation confirms the negative impact of feeds containing pork, which is in line with Altmann et al. [10] who highlighted that more traditionalist consumers may be concerned about the use of animal-based protein meals in animal feed and may, therefore, want to avoid choosing chicken breasts produced using such protein meals as feed. Nevertheless, insect meals do not have a statistically different effect from vegetable feeds on average utility. This is not surprising as the possible use of insects in chicken farming seems to have been internalized by consumers and somewhat accepted [10,30], considering that the willingness to consume meat from animals reared with insects has already been highlighted in recent literature [19,25,27].

One of the most qualifying results of the present work is the relevance of the free-form attribute. The use of clean labels and, in particular, “Free-from” claims has increased strongly in recent years. Many firms have ridden the wave of this trend and exploited “Free-from” clean labels, both to occupy specific market niches and as a tool for strategic competition with other firms and companies [45,56]. Gluten- and lactose-free products have experienced a sudden growth in the market, and their expansion is accompanied by a massive use of “Free-from” claims [46]. The same has been observed for livestock products without antibiotics and/or hormones [57] or the use of palm oil in processed foods [58]. In this case, the use of the claim “free from palm oil” is stimulated in part by the EU Reg. 1169/2011, which requires companies to write the names of specific animal and/or vegetable fats on the label instead of the generic wording “animal fats” or “vegetable oils”. Considering the hostility of consumers toward palm oil due to possible harmful effects on human health and the environment, many companies have abandoned the use of palm oil and used the claim “palm oil free” as leverage to attract consumers concerned about this ingredient [59,60]. 

In the case of new feeds containing animal proteins, a similar mechanism may also be at work. Considering the results of the present research, this risk is particularly high in the case of feeds containing pork meal. Indeed, it has been observed that in both the BWS and the conditional logit estimation, feeds containing pork are associated with particularly negative scores and consistently reduce the average utility. As happened in the case of palm oil, the emergence of feeds containing pork flour could induce other companies to adopt “free-from” claim. This could potentially fuel the ongoing debate on the governance of public and private forms of information regulation, as companies may use different labeling claims to differentiate their products and gain a competitive advantage in the market. 

### Limitations of the Study and Further Developments

The present research has at least three limitations that are worth being acknowledged. One of the most relevant is the small sample size that is restricted to only Italian participants and recruited via the snowball sampling technique, which may limit the generalizability of the findings to larger populations and may not have enough statistical strength to detect important effects or relationships. Secondly, we did not consider any socio-demographic characteristics of the sample as potential explanatory variables. Future research could aim to replicate the findings in a larger and representative sample, as well as investigate potential moderators and mediators of the observed effects, including considering possible differences across gender and age and investigating the role of other socio-demographic characteristics. Finally, the present study did not use any monetary-based measures, such as consumers’ willingness to pay (WTP), to assess consumers’ preferences. WTP is crucial when conducting cost–benefit analysis aiming at determining an optimal investment strategy for food product companies seeking to develop new food process chains. Therefore, future research could benefit from incorporating price-related measures, such as WTP, for product attributes that will better capture the economic trade-offs involved in consumers’ decision making. In this regard, it would also be interesting to investigate how different consumer segments value various product attributes, which could help food companies tailor their marketing strategies to specific groups of consumers. Moreover, a further avenue for this topic could be a comparison between the relevance attached to alternative animal feeds by consumers in developed versus developing countries.

## 5. Conclusions

The present study provides insights into consumers’ preferences for extrinsic attributes and food labeling related to chicken breasts from animals reared with alternative feeds. The findings show that the type of farming and the presence of a “Free-from” claim on the label are the most important attributes for consumers, with the type of feed and price having lower salience. This study also confirms that clean labeling is an important trend for firms’ communication strategies toward consumers, with “Free-from” claims being a particularly appealing attribute. The use of insect-based feeds in chicken farming is somewhat accepted by consumers, while pork-based feeds are rated negatively.

Overall, the results of this study provide useful information for producers and policymakers, highlighting the importance of specific attributes in consumers’ decision making and the potential of clean labeling strategies. The findings can inform marketing and labeling decisions, as well as policies aimed at improving animal welfare, and the development of sustainable feeds for animal nutrition. Specifically, this first study suggests that mandatory labeling that signals the use of PAPs as feeds could have a negative impact on the demand for meat products and decrease in their value; hence, their use in the livestock sector needs to be accompanied by accurate communication strategies.

## Figures and Tables

**Table 1:** Descriptive statistics of the sample (*n* = 205).

	Characteristics	% Sample
Gender	Male	41.5
	Female	58.5
Age	18–34	35.6
	35–54	50.7
	55+	13.7
Education	Below Upper Secondary	6.8
	Upper Secondary	49.8
	Tertiary	43.4
Household members	1	15.6
	2	27.3
	3	33.7
	4	18.5
	5+	4.9
Geographical location	North-East	12.2
	North-West	43.4
	Central	7.3
	Southern + Islands	37.1

**Table 2:** Attributes and levels used to create the best–worst scaling.

Attribute	Levels
Type of feeding	Vegetable fodder
	Pork flour
	Insect flour
Type of rearing	Intensive rearing
	Extensive indoor rearing
	Extensive outdoor rearing
Price	EUR 4.50 (11.26 EUR/kg)
	EUR 3.10 (7.74 EUR/kg)
	EUR 5.90 (14.76 EUR/kg)
Free-from	Antibiotics
	Genetically Modified Organism (GMO)
	Cruelty free

**Table 3:** Best-minus-worst (BW) and standardized best-minus-worst (stdBW) scoring for each attribute level.

Attribute	Levels	Best	Worst	BW	stdBW	# Choices	% Level	% Attribute
Type of feeding	Vegetable fodder	121	52	69	0.113	173	4.7%	22.5%
	Pork flour	34	359	−325	−0.531	393	10.7%	
	Insect flour	81	177	−96	−0.157	258	7.0%	
Type of rearing	Intensive rearing	8	445	−437	−0.714	453	12.4%	31.4%
	Extensive indoor rearing	145	146	−1	−0.002	291	8.0%	
	Extensive outdoor rearing	369	37	332	0.542	406	11.1%	
Price	EUR 4.50 (11.26 EUR/kg)	31	93	−62	−0.101	124	3.4%	17.4%
	EUR 3.10 (7.74 EUR/kg)	103	129	−25	−0.041	233	6.4%	
	EUR 5.90 (14.76 EUR/kg)	39	242	−203	−0.332	281	7.7%	
Free-from	Antibiotics	355	24	331	0.541	379	10.4%	28.6%
	GMO	288	86	202	0.330	374	10.2%	
	Cruelty free	255	40	215	0.351	295	8.1%	

Note: “# Choices” column indicates how many time the level has been selected by participants; it is the result of the Best plus Worst columns. % Level indicates the frequency of selection for each level, while % Attribute is the resultant sum of the % Level.

**Table 4:** Conditional logit model estimates.

Attribute Impacts					
	Coefficient	Coefficient Exponent	Standard Error	*z*-Value	*p*-Value
Type of feeding	−0.247	0.781	0.052	−4.713	0.000
Price	−0.146	0.864	0.053	−2.746	0.006
Free-from	1.049	2.854	0.054	19.145	0.000
**Level Scale Values**					
Vegetable fodder	0.937	-	-	-	-
Pork flour	−1.008	0.365	0.063	−15.907	0.000
Insect flour	0.071	1.074	0.057	1.238	0.216
Intensive rearing	−1.936	−	−	−	−
Extensive indoor rearing	0.207	1.230	0.058	3.577	0.000
Extensive outdoor rearing	1.729	5.637	0.063	27.212	0.000
4.50 EUR (11.26 EUR/kg)	0.040	−	−	−	−
3.10 EUR (7.74 EUR/kg)	0.386	1.471	0.059	6.549	0.000
5.90 EUR (14.76 EUR/kg)	−0.426	0.653	0.059	−7.236	0.000
Antibiotics	0.475	−	−	−	−
GMO	−0.314	0.731	0.057	−5.452	0.000
Cruelty free	−0.161	0.851	0.058	−2.777	0.000
Respondents	205				
Observations	1830				

Note: Vegetable fodder, Intensive rearing, 4.50 EUR (11.26 EUR/kg) and Antibiotics are reference levels.

**Table 5:** Ranking of the BWS profiles included in the survey given the conditional logit model estimates.

BWS Profile	Rank	Attribute Levels			
		Type of Feeding	Type of Rearing	Price	Free-From
7	4	Vegetable fodder	Extensive outdoor rearing	3.10 EUR (7.74 EUR /kg)	GMO
9	15	Insect flour	Extensive outdoor rearing	5.90 EUR (14.76 EUR /kg)	Antibiotics
8	33	Pork flour	Extensive outdoor rearing	4.50 EUR (11.26 EUR /kg)	Cruelty free
4	34	Vegetable fodder	Extensive indoor rearing	5.90 EUR (14.76 EUR /kg)	Cruelty free
5	42	Pork flour	Extensive indoor rearing	3.10 EUR (7.74 EUR /kg)	Antibiotics
6	43	Insect flour	Extensive indoor rearing	4.50 EUR (11.26 EUR /kg)	GMO
1	49	Vegetable fodder	Intensive rearing	4.50 EUR (11.26 EUR /kg)	Antibiotics
3	65	Insect flour	Intensive rearing	3.10 EUR (7.74 EUR /kg)	Cruelty free
2	81	Pork flour	Intensive rearing	5.90 EUR (14.76 EUR /kg)	GMO

## Data Availability

Data will be made available by the corresponding author upon request.
